# Unlocking the complexity of hypoxia non-coding transcriptome landscape of breast cancer

**DOI:** 10.1186/1471-2164-15-S2-P40

**Published:** 2014-04-02

**Authors:** Hani Choudhry, Johannes Schodel, Ashwag Albukhari, Spyros Oikonomopoulos, Syed Haider, Francesca Buffa, Ioannis Ragousis, David R Mole, Adrian L Harris

**Affiliations:** 1 Biochemistry Department, King Abdulaziz University, Jeddah, KSA; 2 Department of Nephrology and Hypertension, Friedrich-Alexander-University, Erlangen-Nuremberg, Germany; 3 The Weatherall Institute of Molecular Medicine, University of Oxford, Oxford, UK; 4 The Wellcome Trust Centre for Human Genetics, University of Oxford, Oxford, UK; 5 Department of Oncology, University of Oxford, Oxford, UK; 6 McGill University and Genome Quebec Innovation Centre, Montreal, Canada; 7 The Henry Wellcome Building for Molecular Physiology, University of Oxford, Oxford, UK

## Background

Transcriptional responses to hypoxia are central to the pathogenesis of many types of cancer. Today, pan-genome analyses of hypoxia have been focused on protein-coding genes, however, the role of non-coding RNAs, in particular long non-coding RNAs (lncRNA) is not well characterized [1].

## Materials and methods

We undertook an integrated pan-genomic analysis of the transcriptional responses to hypoxia in MCF7 breast cancer cells, employing total RNA-seq together with ChIP-Seq for the hypoxia-inducible transcription factor (HIF) and for epigenetic marks of transcriptional activation (RNApol2 and histone H3K4me3).

## Results

Analyses revealed that all classes of RNA are significantly regulated by hypoxia. We found significant numbers of lncRNAs are up-regulated in hypoxia and these are associated with epigenetic marks of increased transcription and HIF binding. We describe a number of hypoxia regulated non-annotated RNA species, including several that are antisense to hypoxia regulated protein-coding RNAs. The most hypoxia up-regulated lncRNA was NEAT1. The role of NEAT1 in cancer has not been previously studied. We demonstrate that NEAT1 induction is common in breast cancer cell lines and xenograft models. Finally, selected hypoxia regulated lncRNAs are analysed in a large cohort of breast cancers (n=2000) and found to be associated with poor clinical outcome and clinicopathological features.

## Conclusions

Our findings extend knowledge of the hypoxic transcriptional response into the spectrum of non-coding transcripts. These HIF-regulated non-coding transcripts have the potential to act as biomarkers for breast cancer as well as potential novel therapeutic targets.
